# Long Duration of Ground Motion in the Paradigmatic Valley of Mexico

**DOI:** 10.1038/srep38807

**Published:** 2016-12-09

**Authors:** V. M. Cruz-Atienza, J. Tago, J. D. Sanabria-Gómez, E. Chaljub, V. Etienne, J. Virieux, L. Quintanar

**Affiliations:** 1 Instituto de Geofísica, Universidad Nacional Autónoma de México, Mexico; 2 Facultad de Ingeniería, Universidad Nacional Autónoma de México, Mexico; 3 Escuela de Física, Universidad Industrial de Santander, Bucaramanga, Colombia; 4 Univiversité de Grenoble Alpes, ISTerre, F-38058 Grenoble, France; 5 CNRS, ISTerre, F-38058 Grenoble, France; 6 ARAMCO, Advanced Research Center, Saudi Arabia

## Abstract

Built-up on top of ancient lake deposits, Mexico City experiences some of the largest seismic site effects worldwide. Besides the extreme amplification of seismic waves, duration of intense ground motion from large subduction earthquakes exceeds three minutes in the lake-bed zone of the basin, where hundreds of buildings collapsed or were seriously damaged during the magnitude 8.0 Michoacán earthquake in 1985. Different mechanisms contribute to the long lasting motions, such as the regional dispersion and multiple-scattering of the incoming wavefield from the coast, more than 300 km away the city. By means of high performance computational modeling we show that, despite the highly dissipative basin deposits, seismic energy can propagate long distances in the deep structure of the valley, promoting also a large elongation of motion. Our simulations reveal that the seismic response of the basin is dominated by surface-waves overtones, and that this mechanism increases the duration of ground motion by more than 170% and 290% of the incoming wavefield duration at 0.5 and 0.3 Hz, respectively, which are two frequencies with the largest observed amplification. This conclusion contradicts what has been previously stated from observational and modeling investigations, where the basin itself has been discarded as a preponderant factor promoting long and devastating shaking in Mexico City.

The seismic response of the Valley of Mexico has been for many years a paradigmatic study case in earthquake seismology and engineering. After the devastation of Mexico City (MC) in 1985, when more than 15,000 people died due to a magnitude 8.0 earthquake beneath the coast of Michoacán, more than 450 km away from the city, scientists have attempted to explain site effects such as the extraordinary amplification of seismic waves and the extremely long duration of ground motion in the sedimentary basin where most of the city is located (Fig. 1). Amplification of ground motion due to local soil conditions is a well-known phenomenon. In the Valley of Mexico, spectral amplification for subduction earthquakes (i.e., with epicentral distances greater than 300 km) at soft-soil sites range from 10 to 50 at frequencies between 0.2 and 0.7 Hz with respect to hard-rock sites^1^,^2^. However, the hard-rock sites also experience large amplifications of about 10 due to regional site effects (quantified from attenuation relationships) associated with the volcanic arc deposits where the valley is embedded^3^,^4^. This means that absolute spectral amplifications in the lake-bed zone of the Valley of Mexico may reach values from 100 to 500, which are probably the largest ever reported worldwide^4^.

## Long-lasting ground motion in the Valley of Mexico

While the amplification of seismic waves in the Valley of Mexico has been satisfactorily explained by regional and local soil conditions^5^,^6^,^7^,^8^,^9^,^10^,^11^,^12^, the physical reasons for the long duration of ground motion remain an open question. Initial efforts addressing this issue considered two-dimensional wave propagation models in small-basin configurations with realistic attenuation properties. Results from these exercises led to conclude that surface-waves trains generated at the edges of the basin^10^,^13^,^14^ suffer a rapid decay as they propagate, and thus to discard this mechanism as a possible explanation for the long seismic records^7^,^15^. This conclusion invoked the existence of regional-scale effects producing the elongation of the incoming wavefield to Mexico City from subduction earthquakes, such as multipathing of seismic waves due to scatterers in the crust and surroundings of the basin^16^,^17^, and seismic energy entrapment in both the accretionary prism near the source region and the Transmexican Volcanic Belt (TMVB)^18^,^19^. The interaction between the incoming wavefield and the local basin conditions may also elongate the ground motion when the resonant frequencies of the basin coincide with the dominant periods of the wavefield^20^.

**Figure 1:**
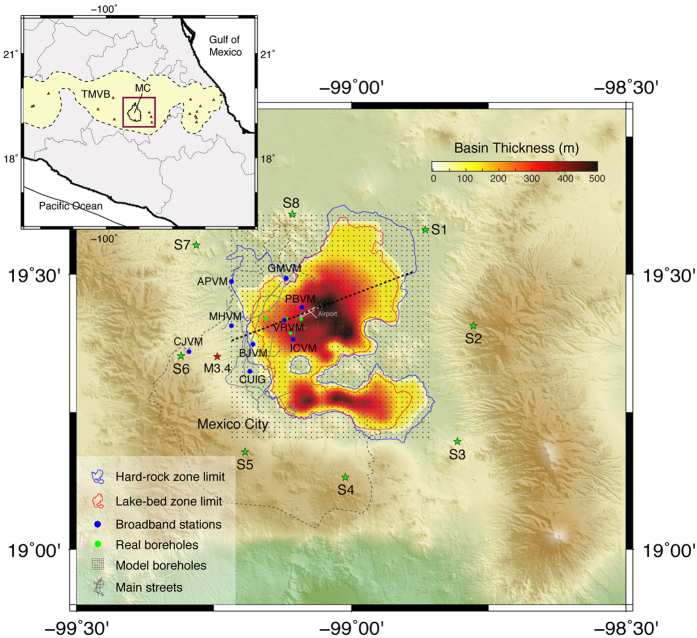
Topographic setting of Mexico City (MC) and the Valley of Mexico. Color scale corresponds to the basin thickness (i.e., the basin contact with the Oligocene volcanics of the Transmexican Volcanic Belt, TMVB). Stars show the epicenters for the vertical body forces applied at the free surface (green) and the magnitude 3.4 earthquake of December 1, 2014 (red). This figure has been created using the Generic Mapping Tools (GMT) Version 5.3.0, http://gmt.soest.hawaii.edu.

**Figure 2:**
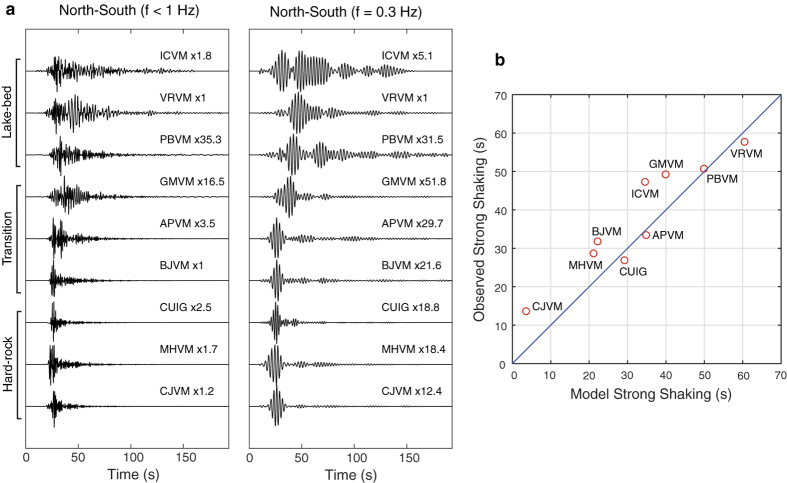
(**a**) Observed velocity seismograms in two different frequency bands at nine broadband seismic stations for a M3.4 earthquake with 4 km depth (see Fig. 1). Records are aligned with the P-wave arrival and scaled with the factors given for each trace. Notice the long seismic records in the lake-bed stations. (**b**) Observed and modeled durations of the strong shaking phase for f < 1 Hz. The corresponding synthetic seismograms are shown in Figure A2.

Although our current understanding of the duration of ground motion in the lake-bed zone of MC is clearly rooted in the nature of the incoming wavefield, the actual effects produced by the sedimentary basin itself have been underestimated. Figure 2a shows the seismic records (f < 1 Hz) of a magnitude 3.4 earthquake that occurred ~4 km below the city on December 1, 2014 (Fig. 1). These unprecedented records were possible thanks to a recently-installed permanent broadband network (blue circles) in the Valley of Mexico operated by the Servicio Sismológico Nacional (SSN). Despite the small magnitude of the event, ground motion in the basin lasted more than two minutes (e.g. at lake-bed sites VRVM, ICVM and PBVM). This can be better appreciated in the band-pass filtered signals at 0.3 Hz, where the long coda is dominated by the harmonic beating widely reported in the literature for subduction earthquakes recorded in MC^16^,^21^. In contrast, this beating is barely present at hard-rock sites such as CUIG and CJVM, where the motion is dominated by a single wave package with duration no longer than 20 s. This observation strongly suggests that ground motion in the lake-bed zone experiences very long durations in the absence of regional-scale effects. Local basin conditions are thus preponderant in the duration of ground shaking across the basin at frequencies where the amplification of seismic waves is the largest. The leading question of this work is thus raised about the mechanisms allowing long-lasting wave propagation within a highly dissipative sedimentary basin. Two main hypotheses may be advanced: (1) the basin incoming wavefield suffers from multiple-scattering even at a local scale, and/or (2) the sedimentary basin itself enhances sustained wave trains generation and efficient propagation.

Though certainly true, hypothesis one does not seem to have first order implications in the duration of ground motion at the lake-bed zone, as revealed by the absence of significant seismic energy in the coda of hard-rock sites (Fig. 2). On the other hand, considering the highly dissipative and fluid saturated sediments that cover large part of the basin (see next section), a plausible idea supporting hypothesis two is the efficient propagation of seismic energy in the deep basin, carried by surface-waves overtones. In this work we examine this argument based on realistic 3D wave propagation modeling to understand whether local soil conditions within the basin may explain the observed long seismic records.

## A basin model for the Valley of Mexico

The Valley of Mexico is located in the southern and volcanically active part of the TMVB (Fig. 1). This region is composed by Oligocene volcanics overlaying Cretaceous limestones. On top of these formations within the valley, there are Miocene volcanics overlain by a ~100 m thick sequence of tuffs or sands, gravel and recent lava flows^11^,^22^, averaging a thickness of ~2 km for the TMVB above the Cretaceous limestones^12^,^23^. Geotechnically speaking, this geologic setting corresponds to the hill zone of the valley (region outside the blue contour in Fig. 1), which may be considered hard-rock sites (e.g. CUIG, MHVM and CJVM). The stratigraphy of the MC basin is essentially the same as in the hill zone except for the absence of recent lava flows and the presence of clays with high water content of 10 to about 100 m thickness^24^,^25^. The basin is geotechnically known as the lake-bed zone of the valley (region within the red contour in Fig. 1) and it is where the largest amplification of ground motion is observed. The transition region in between the lake-bed and the hard-rock zones is mainly composed by alluvial deposits. The composition and thickness of the surficial clay deposits changes laterally within the basin^8^,^22^. However, shear-wave speed measurements on core samples from different boreholes in the lake-bed zone show extremely low values in these deposits (i.e. 30–100 m/s), with an average thickness of about 50 m^24^. Laboratory tests^26^ and field estimates^27^ also show that the clays are highly dissipative, with very low shear Q values ranging from 10 to 50. These observations led to a four-layer velocity model for the basin with very high Vp/Vs values on top to explain experimental data from several earthquakes^28^. We adopt this model for the lake-bed zone in our calculations (Figure A1a and Table A1).

Observations within the basin show that depths (H) to the deep, geotechnically consistent deposits are proportional to the natural vibration periods (T_0_) of the sites (i.e., H = (β/4)*T_0_)^3^. These periods are thus proportional to the thickness of the surficial clay layers that we have assumed constant in our model. However, to confine the underlying deep-basin deposits in depth, from a large data set of natural vibration periods across the basin^2^,^29^ and assuming an average shear-wave speed (β) of 400 m/s, we generated the bed-rock geometry shown in Fig. 1. This interface represents, in our model, the contact between the basin deposits and the Oligocene volcanics of the TMVB (i.e., fourth interface in Figure A1a). A cross section of our basin model along the dotted line of Fig. 1 is shown later in section “Dominance of surface waves overtones”. Regarding the crustal structure surrounding the basin, we adopted a 1D model determined from the inversion of receiver functions at the CUIG site^30^, which includes a relatively low-velocity layer on top, associated with the ~2 km thick TMVB (see Figure A1a and Table A1). To minimize numerical errors, the interfaces of the model were vertically homogenized before discretizing the model by averaging the S- and P-slownesses and densities^31^ (circles in Figure A1a). The homogenization length is 50 m, which is about half of the minimum wavelength in the surficial clay layers. Using the computational method introduced in the next section we calibrated, by trial and error, the attenuation properties of the seismic model so that the durations of the intense phases of ground motions (i.e., time between 5% and 95% of the Arias intensity)^32^ observed for the M3.4 earthquake in nine stations are similar to those predicted by our model assuming a 4 km depth reverse faulting below the epicenter (Figs 2b and A2), as suggested by the first P-wave arrivals. These properties, which are in accordance with laboratory and field measurements^26^,^27^, are such that Qs = 0.3*Vs for Vs < 400 m/s and Qs = 0.1*Vs otherwise, with Qp = 2*Qs everywhere (Table A1).

## Computational method for viscoelastic wave propagation

Simulating the propagation of seismic waves in extreme sedimentary basins represents a big challenge. In our seismic model for the Valley of Mexico, the S-wavelength at 1 Hz shortens from 4.8 km in the deep crust to only 50 m in the top layer of the basin during propagation. To obtain an accurate solution of the elastodynamic equations governing the propagating waves with such modulation, the numerical scheme must handle powerful capabilities to sample the wavefield efficiently in the whole simulation domain. For this reasons we have developed an hp-discontinuous Galerkin finite element method (DG-FEM) called GEODG3D that handles both unstructured domain decompositions (h-adaptivity) and different approximation orders per element in space (p-adaptivity)^33^,^34^,^35^. GEODG3D is an extension for viscoelastic wave propagation of a method previously introduced for elastic waves^33^. It solves the velocity-stress formulation of the visco-elastodynamic equations in three dimensions with rock quality factors, Qs and Qp, chosen to be nearly constant in the frequency range of interest (i.e., f < 1 Hz). For a detailed description of the viscoelastic model and the DG-FEM see Methods.

**Figure 3:**
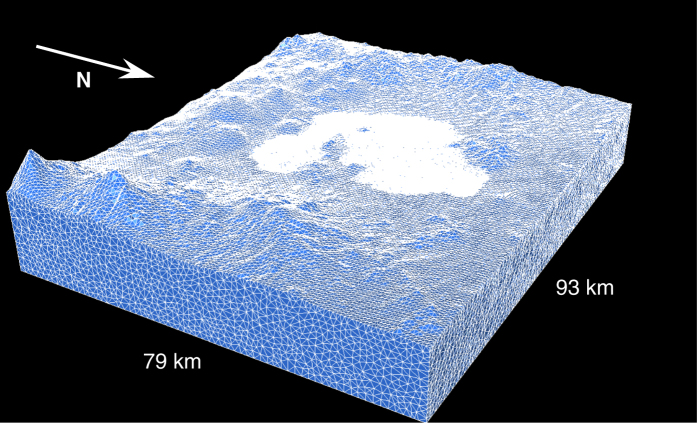
Top ~10 km of the unstructured tetrahedral mesh used in the study. Notice that the elements honor the basin geometry and the actual topography of the terrain (compare with Fig. 1). The mesh considered for the simulations reaches 50 km depth. This figure has been created using the TetView Linux software Version 1.0, http://wias-berlin.de/software/tetgen/tetview.html.

To maximize the integration time step imposed by the Courant stability criterion, GEODG3D locally adapts the elements’ approximation order depending on both the elastic properties of the medium and the size of the tetrahedra (p-adaptivity) (Figure A3a). For decomposing the simulation domain in tetrahedral elements, we followed a meshing strategy that guarantees the same numerical accuracy across the whole domain^33^. Given a maximum resolvable frequency (1 Hz in this work) and starting from a regular coarse mesh, the strategy iteratively refines the elements until the accuracy criterion (i.e., 3 elements per minimum wavelength; Figure A5) is satisfied locally in at least 99.8% of the elements (h-adaptivity) (Figure A3b). Figure 3 shows the resulting mesh for the upper part of the simulation domain, where the elements are clearly adapted to both the actual topography and the extremely low velocities of the basin (compare with Fig. 1). Numerical verification (Figure A4) and convergence analysis (Figure A5) of the GEODG3D viscoelastic solver have been thoroughly done^35^, finding excellent results for different international benchmark problems (see Section 3 of Methods). Table A2 provides useful numbers related to the tetrahedral mesh and discretization parameters used in all simulations of this study.

## Dominance of surface waves overtones

**Figure 4:**
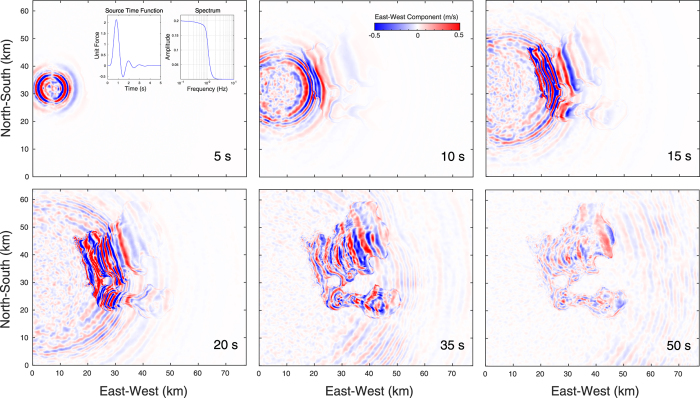
Snapshots of the Green’s function for the vertical body force S6 (see Fig. 1) described by the inset time history with flat spectrum up to 1 Hz. Notice the topographic scattering, the generation and propagation of wave trains at different speeds within the basin, and their multiple diffractions. This figure has been created using the Matlab software Version R2016a, http://www.mathworks.com/.

Observational evidence for the dominance of surface waves overtones in the Valley of Mexico shows that peak displacements in the lake-bed zone between 0.3–0.5 Hz at different borehole depths (green circles in Fig. 1) for several subduction earthquakes are in accordance with theoretical eigenfunctions for the Rayleigh-waves first overtones in the basin model of Table A1^28^. These observations, which are shown later in section “Dominance of surface waves overtones”, reveal that seismic energy barely decays with depth. Furthermore, surface waves dispersion diagrams generated from the correlation of ambient noise in the lake-bed zone also show the overwhelming dominance of first overtones^36^. To understand the physical reason explaining these observations and to quantify the implications in terms of ground motion duration, we first analyzed Green’s functions in our 3D model of the Valley of Mexico (Fig. 3) for eight vertical forces applied at the free surface around the basin (green stars in Fig. 1). The sources radiation thus corresponds to P- and S-waves followed by a dominant Rayleigh train. In order to quantify the effect of attenuation, we performed the simulations for both the elastic and the viscoelastic cases up to 220 s in the UNAM supercomputer Miztli. Velocity snapshots for the viscoelastic simulation with source S6 are shown in Fig. 4, where amplification, diffraction and generation of surface waves at the basin edges are clearly observed.

**Figure 5:**
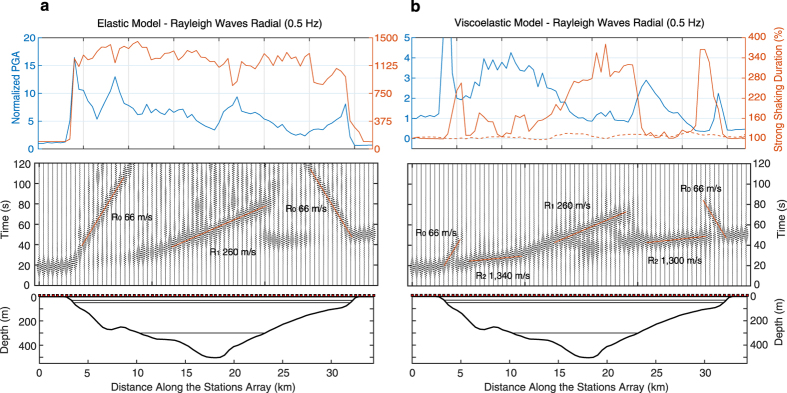
Seismic sections of the radial-component at 0.5 Hz for source S6 along the linear array shown in Fig. 1 for the elastic (**a**) and viscoelastic (**b**) models. A cross section of the basin model is shown at the bottom. On top, peak ground accelerations (PGAs, radial component) along the array (blue) normalized by the value at the station with smallest epicentral distance. Durations of the strong shaking phase along the array (orange) as percentages of the duration of the incoming wavefield (i.e., the duration measured at the station with smallest epicentral distance). As a reference, the dashed line indicates the durations considering only the 1D regional structure (i.e., in the absence of the basin).

Figure 5 shows normalized seismic profiles at 0.5 Hz with 500 m spacing for source S6 along the dashed line of Fig. 1. In the elastic case (Fig. 5a), three main pulses are observed. Two of them propagate from the basin edges with speed of ~66 m/s, and the other emerges at ~10 km of the array with a speed of 260 m/s. Considering the Rayleigh waves group-velocity dispersion curves for shallow and deep basin locations (circles in Figure A1b), speeds clearly correspond to the fundamental mode (R0) and first overtones (R1), respectively. It is striking that even in the absence of attenuation, the first overtone dominates in the deep basin (i.e., between 10 and 23 km along the array). Unlike the elastic case, attenuation rapidly dissipates the fundamental mode and makes the overtones dominant along almost the whole array in the viscoelastic simulation (Fig. 5b). The most prominent wave train in the shallow basin regions propagates with the speed of the bedrock fundamental mode (i.e., ~1,300 m/s, Figure A1b). This means that the incident R0 suffers a mode conversion to become the second overtone (R2) when transduced into the basin, and that such overtone dominates the ground motion at shallow regions thanks to the rapid dissipation of the basin fundamental mode, R0. Around 12 km along the array, the R1 becomes dominant when surface waves propagate from shallow to deeper parts of the basin, revealing the strong influence of the deep sediments.

**Figure 6:**
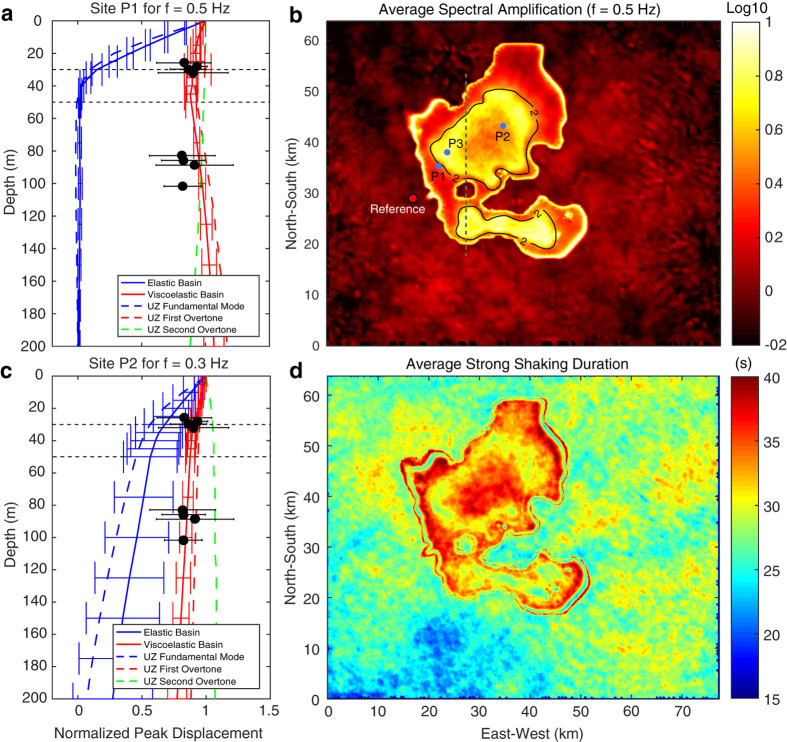
(**a,c**) Comparison of average eigenfunctions for the 8 sources with standard deviation bars for both elastic (blue solid) and viscoelastic (red solid) simulations at two representative sites, P1 and P2, and different frequencies. Dashed lines show theoretical eigenfunctions for the vertical component of Rayleigh waves in the model of Figure A1a (Table A1) for the fundamental mode (blue) and the first (red) and second (green) overtones. Normalized peak vertical displacements observed in different boreholes (green dots in Fig. 1) are shown with black circles and error bars (after Shapiro *et al*., 2001). (**b**) Fourier spectral amplifications (geometric mean of both horizontal components) at 0.5 Hz with respect to the CUIG site (Fig. 1) averaged for the 8 sources. The black contour corresponds to the 2 s dominant-period. (**d**) Duration of the strong shaking phase for f < 1 Hz averaged for the 8 sources. This figure has been created using the Matlab software Version R2016a, http://www.mathworks.com/.

A systematic analysis is necessary to conclude that overtones dominate the ground motion in the whole basin model. We thus analyzed the wavefields from the eight sources at a regular network of boreholes with 1 km spacing (gray dots in Fig. 1). From the seismograms at the network we computed and averaged synthetic eigenfunctions of Rayleigh waves for all sources in each borehole. To do so we normalized vertical displacements along the boreholes by the corresponding peak values at the free surface and at the same absolute time^28^. Figure 6a and c show the average eigenfunctions with standard deviation bars for both the elastic (blue solid lines) and viscoelastic (red solid lines) simulations at two representative sites, P1 and P2, and different frequencies. In the unrealistic elastic simulations and shallow basin regions (i.e., <250 m deep; e.g. at site P1), the energy at 0.5 Hz decays very rapidly with depth (see Figure A6a) and, consequently, the corresponding eigenfunctions fit the expected shape for the fundamental mode (dashed blue line in Fig. 6a). In contrast, in the realistic viscoelastic simulations the energy efficiently travels in depth so that the eigenfunctions fit the theoretical shape for the first and second overtones (dashed red and green lines). Something similar happens in the deep basin at 0.3 Hz (e.g., at site P2), where the elastic and viscoelastic eigenfunctions follow the expected shapes for the fundamental and first overtone, respectively (dashed lines in Fig. 6c and Figure A6b). Although it is difficult to identify propagating pulses in the seismic profiles at 0.3 Hz (Figure A7), eigenfunctions at shallow regions reveal that ground motion is dominated by the first and/or second overtones in both the elastic and viscoelastic cases (Figure A9d). In deeper locations and 0.5 Hz, while the viscoelastic simulations are clearly dominated by the first and/or second overtones, variability of the elastic eigenfunctions reveals a contested dominance between the fundamental and higher modes (Figure A9b). In conclusion, ground motion between 0.3 and 0.5 Hz in the viscoelastic model are dominated, across the whole basin, by surface-waves overtones as data from real boreholes suggest^29^ (black circles in Fig. 6a,c). The strong attenuation in the top clay layers is responsible for this propagation regime by dissipating the fundamental mode (Figure A6). RMS differences of the elastic and viscoelastic averaged eigenfunctions in the whole borehole network are shown in Figures A9a and A9c, where the shallow and deep regions of the basin are clearly distinguished by yellow colors. In those regions and frequencies, the attenuation plays a major role promoting the overtones dominance.

## Implications for the duration of ground motion

Since the attenuation decreases with depth, seismic energy in the deep basin carried by overtones can propagate long distances. Basin-transduced surface waves and generation of wave trains at the basin edges, in addition to the wavefield dispersion and diffraction across the 3D structure, should then elongate the duration of ground motion. The top panels of Figs 5 and A7 show durations of the strong shaking phase for Rayleigh waves along the seismic profile for source S6. In the realistic viscoelastic model, durations grow as the basin becomes deeper, reaching values of 170–280% and 290–500% of the incoming field duration at 0.5 and 0.3 Hz, respectively. A similar situation is found for Love waves (transverse component) as shown in Figure A8, where relative durations vary as 160–280% at 0.5 Hz and 200–500% at 0.3 Hz along almost the whole array. Results for Love waves where obtained applying a 1.5 km depth double-couple strike-slip point source at location S6. In shallow regions (i.e. <300 m deep), peak ground accelerations (PGAs) are the largest, although significant amplification also occurs far from the source, between 22 and 28 km of the array for Rayleigh waves. As expected, amplification of single wave packages in the shallow basin region shortens the strong shaking duration. This is clear in both figures from the anticorrelation of PGA and durations along the profile. Horizontal spectral amplifications (geometric mean of both horizontal components) at 0.5 Hz with respect to the CUIG site averaged for all sources reach values larger than 10 along two ring-like regions encompassed by the 2 s dominant-period contour (Fig. 6b). These estimates are in qualitative agreement with empirical values of spectral accelerations at the same frequency^37^ and suggest that regions with largest amplification may be explained by the geometry of the deep basin. Significantly larger amplifications (up to 25) are found at the lake-bed representative site P3 (Fig. 6b) around ~1.8 and ~3.2 s (Figure A10), which are two periods with similar amplification levels (with respect to CUIG) for subduction earthquakes at near by locations^2^. These results give confidence in our model predictions in terms of amplification patterns in the valley.

Average durations of horizontal strong shaking in regions with large amplification are relatively small for the reason explained above (Fig. 6d). However, durations in most regions of the basin exceed 40 s as observed in the lake-bed stations for the M3.4 earthquake (Fig. 2). Although much smaller in amplitude, ground motions at hard-rock are also long due to the scatter effect of the basin in the opposite side of the source. This is clearly seen in Figure A11, where ground motion duration inside and outside the basin is the same within the shadow-like region, proving that seismic energy recorded at hard-rock sites does not necessarily correspond to the incoming wavefield of the basin, as suggested by several authors^5^,^8^,^16^. Our simulations show that duration of ground motion is remarkably lengthened at frequencies with the largest amplification in the lake-bed (i.e., between ~0.2 and ~0.7 s) (Figs 5, A7 and A8). Long shaking duration at these frequencies may cause large structural damage in Mexico City due to the accumulation of yielding cycles that lengthen the natural vibration periods of the structures. Such mechanism makes these periods to approach those of the soil promoting structural failure, as observed during the devastating 1985 earthquake where more than three hundred 9–12 story, relatively small buildings collapsed^38^,^39^.

In conclusion, our results demonstrate that waves overtones dominate the ground motion in the lake-bed zone of the Valley of Mexico and that this propagation regime strongly contributes to the elongation of intense shaking (i.e., duration of both Rayleigh and Loves waves longer than 170% and 290% of the incoming field duration at 0.5 and 0.3 Hz, respectively) at frequencies where the largest amplification is observed. The bedrock fundamental mode is transduced into the basin and converted into overtones (first and second modes) that dominate the ground motion. The structure of the deep basin is responsible for this mechanism, proving that local basin conditions remarkably increase the duration of strong motion in the lake-bed despite the highly dissipative surficial sediments. Our results imply that duration of the incoming wavefield from subduction earthquakes should be significantly shorter than the observed duration in the lake-bed zone. This conclusion contradicts what has been previously stated from observational and theoretical studies considering the ground motion at hard-rock sites as the basin incoming wavefield. The contradiction can be explained if the seismic coda at those sites is dominated by multiple-scattered local waves generated at the basin, as suggested by our simulations.

## Additional Information

**How to cite this article**: Cruz-Atienza, V. M. *et al*. Long Duration of Ground Motion in the Paradigmatic Valley of Mexico. *Sci. Rep.* **6**, 38807; doi: 10.1038/srep38807 (2016).

**Publisher's note:** Springer Nature remains neutral with regard to jurisdictional claims in published maps and institutional affiliations.

## Supplementary Material

Supplementary Information
