# Supplementary material for: Long Duration of Ground Motion in the Paradigmatic Valley of Mexico
Source: Sci Rep. 2016 Dec 9;6:38807. doi: 10.1038/srep38807 (PMC5146958; doi:10.1038/srep38807)
Supplement: Supplementary Information [file srep38807-s1.pdf]

Supplementary Information

of "Long Duration of Ground Motion in the Paradigmatic Valley of Mexico"

V.M. Cruz-Atienza, J. Tago, J.D. Sanabria-Gómez, E. Chaljub,
V. Etienne, J. Virieux and L. Quintanar

August 26, 2016

In this section we introduce the mathematical and computational models used to simulate the wave propagation in the Valley of Mexico. First, we introduce the model for a viscoelastic rheology and how we couple the corresponding constitutive relationship with the equation of motion to get the hyperbolic system describing the viscoelastic wave propagation. Then, we introduce the system discretization following a discontinuous Galerkin strategy, and present the computational model verification and convergence analysis based on different international benchmark problems. We also include all tables and supplementary figures referred in the main text of the manuscript.

1 Viscoelastic model for waves propagation

The stress-strain constitutive relationship in an isotropic viscoelastic medium can be defined as

$$\begin{aligned}\sigma_{ij}(t) &= \delta_{ij}\delta_{kl}\int_{-\infty}^{t}\lambda(t-\tau)\partial_t\epsilon_{kl}(\tau)d\tau \\ &\quad +(\delta_{ik}\delta_{jl}+\delta_{il}\delta_{jk})\int_{-\infty}^{t}\mu(t-\tau)\partial_t\epsilon_{kl}(\tau)d\tau, \end{aligned} \quad (1)$$

where $\sigma_{ij}(t)$ is the stress tensor, $\lambda(t)$ and $\mu(t)$ are the Lamé relaxation functions and $\epsilon_{kl}(t)$ is the strain tensor.

To avoid the unaffordable computation of the time convolutions in equation (1), for each Lamé relaxation function we will model the viscoelastic rheology as a *Generalized Maxwell Body* (GMB-EK) with n *Maxwell Bodies* (MB) and 1 *Hooke Body* (HB) connected in parallel[4]. In the frequency domain, the relaxation functions can be written as

$$\lambda(\omega) = \lambda_U\left(1-\sum_{l=1}^{n}Y_l^{\lambda}\frac{\omega_l}{\omega_l+i\omega}\right) \quad (2)$$

$$\mu(\omega) = \mu_U\left(1-\sum_{l=1}^{n}Y_l^{\mu}\frac{\omega_l}{\omega_l+i\omega}\right), \quad (3)$$

where λ_U and μ_U are the unrelaxed Lamé parameters that correspond to the instantaneous elastic response of the viscoelastic material, Y_l^{λ} and Y_l^{μ} are the anelastic coefficients and ω_l are the relaxation frequencies for the lth MB. The anelastic coefficients, Y_l^{λ} and Y_l^{μ}, gather some physical properties of the propagation medium[11] and its computation will be explain in Section 1.1.

We use the inverse Fourier transformation to express the Lamé relaxation functions (equations (2) and (3)) in the

time domain, so that convolutions in equation (1) may be written as:

$$\int_{-\infty}^{t} \lambda(t-\tau)\partial_t \epsilon_{kl}(\tau) d\tau = \lambda_U \epsilon_{kl} - \lambda_U \sum_{m=1}^{n} Y_m^{\lambda} \zeta_m^{kl}(t) \quad (4)$$

$$\int_{-\infty}^{t} \mu(t-\tau)\partial_t \epsilon_{kl}(\tau) d\tau = \mu_U \epsilon_{kl} - \mu_U \sum_{m=1}^{n} Y_m^{\mu} \zeta_m^{kl}(t), \quad (5)$$

where we define the anelastic functions as

$$\zeta_m^{kl}(t) = \omega_m \int_{-\infty}^{t} \epsilon_{kl}(\tau) \exp^{-\omega_m(t-\tau)} d\tau \qquad m = 1, \cdots, n \quad k, l \in \{1,2,3\}, \quad (6)$$

and, because of the exponential term in equation (6), the time evolution of the anelastic functions can be associated with their own ODE[8]:

$$\partial_t \zeta_m^{kl}(t) + \omega_m \zeta_m^{kl}(t) = \omega_m \epsilon_{kl}(t) \qquad m = 1, \cdots, n \quad k, l \in \{1,2,3\}. \quad (7)$$

By substituting equations (4) and (5) into equation (1) our constitutive relationship becomes

$$\begin{aligned} \sigma_{ij}(t) &= \delta_{ij}\delta_{kl}\lambda_U \epsilon_{kl}(t) + (\delta_{ik}\delta_{jl} + \delta_{il}\delta_{jk})\mu_U \epsilon_{kl}(t) \\ &\quad - \sum_{m=1}^{n} (\delta_{ij}\delta_{kl}\lambda_U Y_m^{\lambda} \zeta_m^{kl}(t) + (\delta_{ik}\delta_{jl} + \delta_{il}\delta_{jk})\mu_U Y_m^{\mu} \zeta_m^{kl}(t)). \end{aligned} \quad (8)$$

To avoid having the physical properties involved in the fluxes computation[1] (see Section 2), we define the stress vector as $\vec{\sigma} = (\omega, \omega', \omega'', \sigma_{xy}, \sigma_{xz}, \sigma_{yz})^t$ with $\omega = \frac{1}{3}(\sigma_{xx}+\sigma_{yy}+\sigma_{zz})$, $\omega' = \frac{1}{3}(2\sigma_{xx}-\sigma_{yy}-\sigma_{zz})$ and $\omega'' = \frac{1}{3}(-\sigma_{xx}+2\sigma_{yy}-\sigma_{zz})$. This change of variable allows to express equation (8) in the following matrix form as

$$\Lambda \vec{\sigma} = \sum_{\theta \in \{x,y,z\}} \partial_\theta \mathcal{N}_\theta \vec{u} - \sum_{l=1}^{n} \mathcal{A}_l \vec{\zeta_l} \quad (9)$$

where $\vec{u} = (u_x, u_y, u_z)^t$ is the displacement vector and $\vec{\zeta_l} = (\zeta_l^{xx}, \zeta_l^{yy}, \zeta_l^{zz}, \zeta_l^{xy}, \zeta_l^{xz}, \zeta_l^{yz})^t$ is the anelastic function vector for the lth MB. Matrix $\Lambda = \text{diag}[3/(3\lambda_U + 2\mu_U), (3/2\mu_U), 3/(2\mu_U), 1/\mu_U, 1/\mu_U, 1/\mu_U]$, which gathers the physical properties of the medium, is given by the unrelaxed Lamé parameters, λ_U and μ_U, and $\mathcal{N}_\theta$ are constant real matrices defined as

$$\mathcal{N}_x = \begin{pmatrix} 1 & 2 & -1 & 0 & 0 & 0 \\ 0 & 0 & 0 & 1 & 0 & 0 \\ 0 & 0 & 0 & 0 & 1 & 0 \end{pmatrix}^T \quad \mathcal{N}_y = \begin{pmatrix} 0 & 0 & 0 & 1 & 0 & 0 \\ 1 & -1 & 2 & 0 & 0 & 0 \\ 0 & 0 & 0 & 0 & 0 & 1 \end{pmatrix}^T \quad \mathcal{N}_z = \begin{pmatrix} 0 & 0 & 0 & 0 & 1 & 0 \\ 0 & 0 & 0 & 0 & 0 & 1 \\ 1 & -1 & -1 & 0 & 0 & 0 \end{pmatrix}^T.$$

Matrix $\mathcal{A}$, associated with the anelastic term of equation (8), is given by

$$\mathcal{A}_l = \begin{bmatrix} \mathcal{A}_l^1 & \mathcal{A}_l^1 & \mathcal{A}_l^1 & 0 & 0 & 0 \\ 2\mathcal{A}_l^2 & -\mathcal{A}_l^2 & -\mathcal{A}_l^2 & 0 & 0 & 0 \\ -\mathcal{A}_l^2 & 2\mathcal{A}_l^2 & -\mathcal{A}_l^2 & 0 & 0 & 0 \\ 0 & 0 & 0 & 2\mathcal{A}_l^2 & 0 & 0 \\ 0 & 0 & 0 & 0 & 2\mathcal{A}_l^2 & 0 \\ 0 & 0 & 0 & 0 & 0 & 2\mathcal{A}_l^2 \end{bmatrix}, \quad (10)$$

where $\mathcal{A}_l^1 = \frac{3\lambda_U Y_l^{\lambda} + 2\mu_U Y_l^{\mu}}{3\lambda_U + 2\mu_U}$ and $\mathcal{A}_l^2 = Y_l^{\mu}$.

To obtain the first part of the hyperbolic system of partial differential equations governing the propagation of viscoelastic waves, we just applied the time derivative to equation (9):

$$\Lambda \partial_t \vec{\sigma} = \sum_{\theta \in \{x,y,z\}} \partial_\theta \mathcal{N}_\theta \vec{v} - \sum_{l=1}^{n} \mathcal{A}_l \vec{\xi}_l, \tag{11}$$

where $\vec{v} = \partial_t \vec{u} = (v_x, v_y, v_z)^t$ is the velocity vector, and $\vec{\xi}_l = \partial_t \vec{\zeta}_l = (\xi_l^{xx}, \xi_l^{yy}, \xi_l^{zz}, \xi_l^{xy}, \xi_l^{xz}, \xi_l^{yz})^t$, is the time derivative of the anelastic functions vector $\vec{\zeta}_l$ that for simplicity, we will still call the anelastic function vector. Then the ODE associated with $\vec{\xi}_l$ is given by

$$\partial_t \vec{\xi}_l + \omega_l \vec{\xi}_l = \omega_l \sum_{\theta \in \{x,y,z\}} \partial_\theta \mathcal{O}_\theta \vec{v}, \tag{12}$$

where

$$\mathcal{O}_x = \begin{bmatrix} 1 & 0 & 0 & 0 & 0 & 0 \\ 0 & 0 & 0 & 0.5 & 0 & 0 \\ 0 & 0 & 0 & 0 & 0.5 & 0 \end{bmatrix}^T \mathcal{O}_y = \begin{bmatrix} 0 & 0 & 0 & 0.5 & 0 & 0 \\ 0 & 1 & 0 & 0 & 0 & 0 \\ 0 & 0 & 0 & 0 & 0 & 0.5 \end{bmatrix}^T \mathcal{O}_z = \begin{bmatrix} 0 & 0 & 0 & 0 & 0.5 & 0 \\ 0 & 0 & 0 & 0 & 0 & 0.5 \\ 0 & 0 & 1 & 0 & 0 & 0 \end{bmatrix}^T. \tag{13}$$

To complete the hyperbolic system and keep track of the time-varying velocity field, we incorporate the equation of motion. This equation can be written in matrix form[1] as

$$\rho \partial_t \vec{v} = \sum_{\theta \in \{x,y,z\}} \partial_\theta \mathcal{M}_\theta \vec{\sigma} + \vec{f}, \tag{14}$$

where ρ is the medium density, $\vec{f}$ is the external force vector and $\mathcal{M}_\theta$ are constant real matrices defined as

$$\mathcal{M}_x = \begin{pmatrix} 1 & 1 & 0 & 0 & 0 & 0 \\ 0 & 0 & 0 & 1 & 0 & 0 \\ 0 & 0 & 0 & 0 & 1 & 0 \end{pmatrix} \quad \mathcal{M}_y = \begin{pmatrix} 0 & 0 & 0 & 1 & 0 & 0 \\ 1 & 0 & 1 & 0 & 0 & 0 \\ 0 & 0 & 0 & 0 & 0 & 1 \end{pmatrix} \quad \mathcal{M}_z = \begin{pmatrix} 0 & 0 & 0 & 0 & 1 & 0 \\ 0 & 0 & 0 & 0 & 0 & 1 \\ 1 & -1 & -1 & 0 & 0 & 0 \end{pmatrix}.$$

Thus, equations (11) and (14) constitute the hyperbolic system fully describing the viscoelastic wave propagation.

1.1 Computation of the anelastic coefficients

The computation of the anelastic coefficients, Y_l^λ and Y_l^μ, in equations (2) and (3) is done such that the quality factors, Q_φ, for $\varphi \in \{\lambda, \mu\}$, are approximated over a frequency range of interest. The inverse of the quality factor is defined for each Lamé relaxation function (equations (2) and (3)) as

$$Q_\varphi^{-1}(\omega) = \frac{\mathrm{Im}\varphi(\omega)}{\mathrm{Re}\varphi(\omega)} = \sum_{l=1}^{n} \frac{\omega_l \omega + Q^{-1}(\omega)\omega_l^2}{\omega_l^2 + \omega^2} Y_l^\varphi \qquad \varphi \in \{\lambda, \omega\}. \tag{15}$$

To approximate a nearly constant $Q_\varphi(\omega)$ in a given frequency range, we set the relaxation frequencies ω_l in the frequency range of interest with a logarithmically equidistant spacing[4]. A constant Q has been proved to be a good approximation for most geophysical applications[9]. Nonetheless, the following procedure can also be applied for any frequency dependency of Q[10]. Once the relaxation frequencies are spread along the frequency range, we used a least square method to determine the anelastic coefficients in equation (15) that better fit the function $Q_\varphi(\omega)$.

In practice, seismologists describe the rocks anelastic dissipation through the quality factors Q_α and Q_β associated with the P- and S- waves, respectively. After computing their corresponding coefficients Y_l^α and Y_l^β, we can compute

those related with the Lamé parameters using the transformations

$$Y_l^\lambda = \left(1 + \frac{2\mu}{\lambda}\right) Y_l^\alpha - \frac{2\mu}{\lambda} Y_l^\beta \qquad \text{and} \qquad Y_l^\mu = Y_l^\beta. \tag{16}$$

For our simulations, we have considered three MB (i.e., three relaxation frequencies) to approximate constant Q_α and Q_β in the frequency range [0.01 5.0] Hz, which is a reasonable choice for our modeling purposes. The more relaxation frequencies we consider the better is the approximation of the given function $Q_\varphi(\omega)$. However, it is important to notice that increasing the amount of relaxation frequencies implies a significant increment in the memory storage requirements and computational time.

2 hp-Discontinuous Galerkin method

Before solving the hyperbolic system given by equations (11) and (14), we first need to decompose the physical domain, Ω, into K elements, so that

$$\Omega \simeq \Omega_h = \sum_{i=1}^{K} D_i \tag{17}$$

where each D_i is a straight-sided tetrahedron whose union constitutes a geometrically conforming mesh .

We approximate the velocity and stress vectors in every tetrahedron, D_i $\forall i \in \{1, \ldots, K\}$, using a nodal interpolation[6] as

$$\hat{\vec{v}}_i(\vec{x}, t) = \sum_{j=1}^{d_i} \vec{v}_{i_j}(\vec{x}_j, t)\varphi_{i_j}(\vec{x}) \tag{18}$$

$$\hat{\vec{\sigma}}_i(\vec{x}, t) = \sum_{j=1}^{d_i} \vec{\sigma}_{i_j}(\vec{x}_j, t)\varphi_{i_j}(\vec{x}), \tag{19}$$

where $\vec{x} \in D_i$, t is the time and d_i is the number of nodes supporting the interpolation Lagrangian polynomial basis functions, φ_{i_j}, associated to the j-node located at $\vec{x}_j$.

Using the nodal interpolations (18) and (19), we can apply a discontinuous Galerkin approach[5] to equations (11) and (14) and get

$$\begin{aligned} \rho_i(\mathcal{I}_3 \otimes \mathcal{K}_i)\frac{\vec{v}_i^{n+\frac{1}{2}} - \vec{v}_i^{n-\frac{1}{2}}}{\Delta t} &= -\sum_{\theta\in\{x,y,z\}} (\mathcal{M}_\theta \otimes \mathcal{E}_{i\theta})\vec{\sigma}_i^n \\ &\quad + \frac{1}{2}\sum_{k\in N_i} \left[(\mathcal{P}_{ik} \otimes \mathcal{F}_{ik})\vec{\sigma}_i^n + (\mathcal{P}_{ik} \otimes \mathcal{G}_{ik})\vec{\sigma}_k^n\right] \end{aligned} \tag{20}$$

$$\begin{aligned} (\Lambda_i \otimes \mathcal{K}_i)\frac{\vec{\sigma}_i^{n+1} - \vec{\sigma}_i^n}{\Delta t} &= -\sum_{\theta\in\{x,y,z\}} (\mathcal{N}_\theta \otimes \mathcal{E}_{i\theta})\vec{v}_i^{n+\frac{1}{2}} - \sum_{l=1}^{n}(\mathcal{A}_{i_l} \otimes \mathcal{K}_i)\vec{\xi}_{i_l}^{n+\frac{1}{2}} \\ &\quad + \frac{1}{2}\sum_{k\in N_i} \left[(\mathcal{Q}_{ik} \otimes \mathcal{F}_{ik})\vec{v}_i^{n+\frac{1}{2}} + (\mathcal{Q}_{ik} \otimes \mathcal{G}_{ik})\vec{v}_k^{n+\frac{1}{2}}\right] \end{aligned} \tag{21}$$

where the matrices involved are: the mass matrix

$$(\mathcal{K}_i)_{rj} = \int_{V_i} \varphi_{i_r}\varphi_{i_j} dV \qquad j, r \in [1, d_i],$$

the stiffness matrix

$$(\mathcal{E}_{i\theta})_{rj} = \int_{V_i} (\partial_\theta \varphi_{i_r})\varphi_{i_j} dV \qquad j,r \in [1,d_i] \qquad \theta \in \{x,y,z\},$$

the flux matrices

$$\begin{aligned}(\mathcal{F}_{ik})_{rj} &= \int_{S_{ik}} \varphi_{i_r}\varphi_{i_j} dS \qquad j,r \in [1,d_i] \\ (\mathcal{G}_{ik})_{rj} &= \int_{S_{ik}} \varphi_{i_r}\varphi_{k_j} dS \qquad r \in [1,d_i] \qquad j \in [1,d_k].\end{aligned}$$

and the auxiliary flux matrices

$$\begin{aligned}\mathcal{P}_{ik} &= \sum_{\theta\in\{x,y,z\}} n_{ik_\theta}\mathcal{M}_\theta \\ \mathcal{Q}_{ik} &= \sum_{\theta\in\{x,y,z\}} n_{ik_\theta}\mathcal{N}_\theta,\end{aligned}$$

where $\mathcal{I}_3$ is the 3x3 identity matrix, $\otimes$ represent the tensor product, and n_{ik_θ} is the component along the θ axis of the unit normal vector $\vec{n}_{ik}$ of the element face S_{ik} which points from the i- to the k-element.

The size of these matrices depends on the order of the polynomial basis used for the nodal interpolation. The flux terms of the ith-tetrahedron are computed following a non-dissipative centered scheme with its N_i adjacent elements. Besides, thanks to the change of variable previously introduced, the fluxes of equation (21) do not involve the physical properties of the neighboring elements but only their velocity fields.

In our method, we have implemented P0, P1 and P2 (i.e. constant, linear and quadratic) approximation orders that can be individually assigned to each tetrahedron D_i depending on its characteristic size and medium properties (i.e., p-adaptivity). Staggered time integration is performed through a second-order explicit leap-frog scheme, which allows the alternation of velocities and stresses during computation. The order of approximation used for time integration matches the highest approximation order for the spatial interpolation (*i.e.* P2).

To solve the ODE's governing the anelastic functions (12), we approximate these functions using a nodal interpolation and the same Galerkin approach introduced before for equations (11) and (14), to get

$$\begin{aligned}(\mathcal{I}_6\otimes\mathcal{K}_i)\frac{\vec{\xi}_{i_l}^{n+\frac{1}{2}} - \vec{\xi}_{i_l}^{n-\frac{1}{2}}}{\Delta t} &= -\omega_l\left((\mathcal{I}_6\otimes\mathcal{K}_i)\vec{\xi}_{i_l}^{n-\frac{1}{2}} + \sum_{\theta\in\{x,y,z\}}(\mathcal{O}_\theta\otimes\mathcal{E}_{i\theta})\vec{v}_i^{n-\frac{1}{2}}\right) \\ &\quad +\omega_l\frac{1}{2}\sum_{k\in N_i}\left[(\mathcal{R}_{ik}\otimes\mathcal{F}_{ik})\vec{v}_i^{n-\frac{1}{2}} + (\mathcal{R}_{ik}\otimes\mathcal{G}_{ik})\vec{v}_k^{n-\frac{1}{2}}\right] \end{aligned} \tag{22}$$

where $\mathcal{R}_{ik} = \sum_{\theta\in\{x,y,z\}} n_{ik_\theta}\mathcal{O}_\theta$. It is important to notice that the discontinuous Galerkin approximation used for the ODE's allows us to honour the p-adaptivity of the scheme.

In order to achieve good accuracy for P2 elements, the unstructured model discretization must warranty 3 tetrahedra per minimum wavelength[5] (see Figure A5). On the other hand, the scheme stability is given by an heuristic criterion[7] given by

$$\Delta t < \min_i\left(\frac{1}{2k_i+1}\cdot\frac{2r_i}{\alpha_i}\right) \tag{23}$$

where r_i is the radius of the sphere inscribed in the element indexed by i, α_i is the $P-$wave velocity in the element and k_i is the polynomial degree used in the element.

Our Discontinuous Galerkin Finite Element Method (DG-FEM) (i.e., the GEODG3D code) thus has two main features that make it a very flexible and powerful numerical tool. One is the h-adaptivity, which allows working with unstructured tetrahedral meshes geometrically adapted to the physical properties of the medium and the free surface topography, so that the accuracy criterion is satisfied locally (Figures 3a and A3b). The other is the p-adaptivity, that allows choosing the most convenient order of approximation per tetrahedron to relax as much as possible the stability condition (i.e., to maximize the integration time step). A nice example of p-adaptivity is given in Figure A3a, where the elements right below the basin, which have a very little characteristic size and relatively high wave speeds, are low approximation order (i.e., P1 or P0). This numerical approach was developed in recent years during the PhD thesis of Tago (2012)[12], where more methodological and numerical details are provided.

3 Model verification and convergence

To verify the correctness of the solutions yielded by the GEODG3D code, we solved two international benchmarks problems. Solutions were compared with those from AXITRA[2], a semi-analytical discrete wave number method. The benchmarks correspond to the elastic and viscoelastic versions of the Layer Over an Homogeneous half-space problems, LOH1 and LOH3, respectively[3].

For the LOH3 benchmark, the viscoelastic moduli were exactly the same in both the AXITRA and GEODG3D simulations. This choice allows quantifying approximation errors associated only to the implementation of the attenuation model for a given number of relaxation mechanisms and thus for the same $Q(\omega)$ functions. In this benchmark problem, the top layer is 1000 m thick and the physical properties of the whole model are given in Table A3. We approximated the frequency-independent quality factors Q_α and Q_β with three relaxation mechanisms. Receivers are located in the free surface ($z = 0$) with positions relative to the epicenter ($x \to$ North and $y \to$ East) given in Table A4. A double-couple point source is located 2000 m below the free surface with all components of its moment tensor equal to zero except $M_{xy} = M_{yx}$, with moment value $M_0 = 10^{18}\ Nm$. The Moment rate time history is a Gaussian pulse given by

$$\frac{1}{t_r\sqrt{\pi}} \exp \frac{-(t-t_0)^2}{t_r^2}, . \tag{24}$$

where t is the time, $t_r = 0.05\ s$ is the rise time and $t_0 = 0.25\ s$ is the origin time. The source spectrum is almost flat up to 10 Hz so all the frequencies below have almost the same amplitude. Solutions should be compared up to 5.0 Hz.

All solutions were computed using P2 elements in the physical domain, approximately ten P1 elements in the Convolutional Perfectly Matched Layer (CPML)[5] region and free surface boundary conditions on top of the model. The characteristic size of the tetrahedra used for both the structured and the unstructured meshes was 100 m, as suggested in the benchmarks descriptions[3]. This choice is convenient for our method since the number of elements per minimum wavelength, n_λ, is about three, which corresponds to the accuracy criterion for our method[5] (see Figure A5).

Figure A4 shows the comparison of the three velocity components in the farthest three receivers (*i.e.* located about 32 times the minimum wavelength from the epicenter) using an unstructured mesh. The time series were filtered using a two-pass four-pole Butterworth filter in the frequency band $[1-5]$ Hz. The agreement between solutions is excellent (i.e., error of about 1.2%).

We performed a convergence analysis of the GEODG3D method based on both the elastic (LOH1) and the viscoelastic (LOH3) benchmarks considering structured and unstructured meshes. The Normalized Root Mean Square (NRMS)

function was used to quantify the error between our solutions and the AXITRA reference solutions. This function is given by

$$NRMS(v_\theta^{DG-FEM}, v_\theta^{AXITRA}) = \frac{\sqrt{(\sum_{i=1}^{n}(v_{\theta_i}^{DG-FEM} - v_{\theta_i}^{AXITRA})^2)/n}}{max(v_\theta^{AXITRA}) - min(v_\theta^{AXITRA})}, \tag{25}$$

where n is the length of the seismograms vectors and $\theta \in \{x, y, z\}$. Components with no signals were excluded in the NRMS computation. For the rest of receivers we computed the NRMS in the three velocity components and averaged them to have a single misfit value.

Figure A5 presents NRMS values computed for the following four simulation cases: 1) the LOH1 benchmark with structured mesh, 2) the LOH1 benchmark with unstructured mesh, 3) the LOH3 benchmark with structured mesh and 4) the LOH3 benchmark with unstructured mesh. NRMS values are reported as a function of the number of elements per minimum wavelength, n_λ, associated with the cutoff frequency of 5 Hz. Linear regressions are also plotted in the log-log scale. The resulting slopes give the convergence rates of the solutions with respect to n_λ. For the unstructured viscoelastic case (i.e., for conditions similar to our simulations in the Valley of Mexico) the convergence rate is 2.98.

Four main conclusions detach from Figure A5: 1) viscoelastic solutions are systematically better than the elastic ones no matter we use structured or unstructured meshes; 2) the convergence rate of both viscoelastic and elastic solutions is virtually the same and depends on the kind of mesh we use; 3) the convergence rate is significantly higher in unstructured meshes no matter we solve the elastic or viscoelastic equations (*i.e.* convergence rate about 1.8 times higher); and 4) numerical errors are lower than 2% and 1.2% in structured and unstructured meshes, respectively, provided that $n_\lambda >= 3$ no matter we solve the elastic or viscoelastic equations.

References

1. Benjemaa, M., N. Glinsky-Olivier, V. M. Cruz-Atienza, and J. Virieux, 3-d dynamic rupture simulations by a finite volume method, *Geophys. J. Int.*, *178*(1), doi:10.1111/j.1365-246X.2009.04088.x, 2009.

2. Bouchon, M., and O. Coutant, Calculation of synthetic seismograms in a laterally varying medium by the boundary element-discrete wavenumber method, *Bull. Seismol. Soc. Am.*, *84*, 1869–1881, 1994.

3. Day, S. M., J. Bielak, D. Dreger, R. Graves, S. Larsen, K. Olsen, and A. Pitarka, Test of 3D elastodynamics codes: Final report for lifelines project 1A02, *Tech rept.*, Pacific Earthquake Engineering Research Center, Berkeley, California, 2003.

4. Emmerich, H., and M. Korn, Incorporation of attenuation into time-domain computations of seismic wave fields, *Geophysics*, *52*(9), doi:10.1190/1.1442386, 1987.

5. Etienne, V., E. Chaljub, J. Virieux, and N. Glinsky, An hp-adaptive discontinuous Galerkin finite-element method for 3-D elastic wave modelling, *Geophys. J. Int.*, *183*(2), doi:10.1111/j.1365-246X.2010.04764.x, 2010.

6. Hesthaven, J. S., and T. Warburton (Eds.), *Nodal Discontinuous Galerkin Methods: Algorithms, Analysis and Applications*, 1st ed., 515 pp., Springer, 2008.

7. Kaser, M., and M. Dumbser, A highly accurate discontinuous Galerkin method for complex interfaces between solids and moving fluids, *Geophysics*, *73*(3), 23–35, 2008.

8. Kristek, J., and P. Moczo, Seismic-Wave Propagation in Viscoelastic Media with Material Discontinuities: A 3d Fourth-Order Staggered-Grid Finite-Difference Modeling, *Bull. Seism. Soc. Am.*, *93*, doi:10.1785/0120030023, 2003.

9. Liu, H. P., D. L. Anderson, and H. Kanamori, Velocity dispersion due to anelasticity; implications for seismology and mantle composition, *Geophys. J. Roy. Astr. Soc.*, *47*, 41–58, 1976.

10. Liu, P., and R. J. Archuleta, Efficient Modeling of Q for 3D Numerical Simulation of Wave Propagation, *Bull. Seismol. Soc. Am.*, *96*(4), 1352–1358, 2006.

11. Moczo, P., and J. Kristek, On the rheological models used for time-domain methods of seismic wave propagation, *Geophys. Res. Lett.*, *32*(L01306), doi:10.1029/2004GL021598, 2005.

12. Tago, J., Modelado de la dinámica de la fuente sísmica y de la propagación de ondas viscoelásticas con el método de Galerkin discontinuo, Ph.D. thesis, UNAM, Mexico, 2012.

Table A1 Velocity structure considered in this study. Green shaded layers correspond to those inside the 3D basin geometry shown in Figure 1. Blue shaded layers correspond to the 1D structure where the basin is embedded. Thicknesses indicated with stars correspond to the deepest point of the basin. They vary depending on the basin geometry.

H (km)	Vp (km/s)	Vs (km/s)	Rho (gr/cm^3)	Qp	Qs
0.03	0.8	0.05	2.0	30.0	15.0
0.02	1.2	0.1	2.0	60.0	30.0
0.25	2.0	0.4	2.05	80.0	40.0
0.25*	2.5	0.8	2.05	160.0	80.0
1.42*	2.70	1.56	2.20	312.0	156.0
2.34	5.51	3.18	2.53	636.0	318.0
10.97	6.00	3.46	2.69	692.0	346.0
27.62	6.68	3.86	2.91	772.0	386.0
∞	8.31	4.80	3.43	960.0	480.0

Table A2 Numerical information of a typical viscoelastic simulation. The UNAM supercomputing platform Miztli has 40 Gb Infiniband interconextion and processors Intel Xeon E5-2670 with frequency of 2.6 to 3.3 GHz.

**Maximum resolved frequency**	1 Hz
**Size of the simulation domain**	93.3 x 79.7 x 50.3 km
**Length of the CPML layer**	8 km
**Number of mesh elements**	12.25 million
**Elements within the basin**	97.04 %
**P0 elements**	10.41 %
**P1 elements**	12.85 %
**P2 elements**	76.75 %
**Minimum element size**	0.49 m
**Maximum element size**	737 m
**Integration time step**	0.00036 s
**Number of time steps**	514,995
**Number of parallel processors**	512
**Computing elapsed time**	23.7 hr

Table A3: Medium parameters of the LOH3 benchmark

	α (m/s)	β (m/s)	ρ (kg/m^3)	Q_α	Q_β
layer	4000	2000	2600	120	40
halfspace	6000	3464	2700	180	80

Table A4: Receivers location of the LOH1 and LOH3 benchmarks

Receiver	1	2	3	4	5	6	7	8	9
x (m)	0	0	0	490	3919	7348	577	4612	8647
y (m)	693	5543	10392	490	3919	7348	384	3075	5764

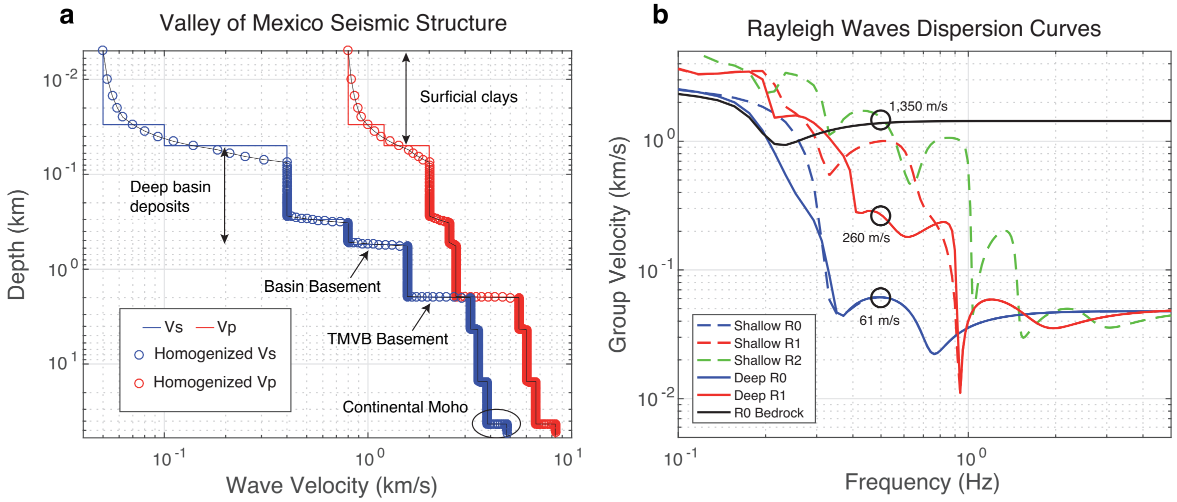

Figure A1 (a) Velocity structure used in the numerical model (see Table A1). Depth of the basin basement varies in space according to the basin thickness shown in Figure 1. Circles show the homogenized velocity structure used for the model discretization. (b) Rayleigh waves dispersion curves for the vertical component of the fundamental (R0, blue) and first overtone (R1, red) at shallow (250 m thick; dashed) and deep (500 m thick; solid) basin sites.

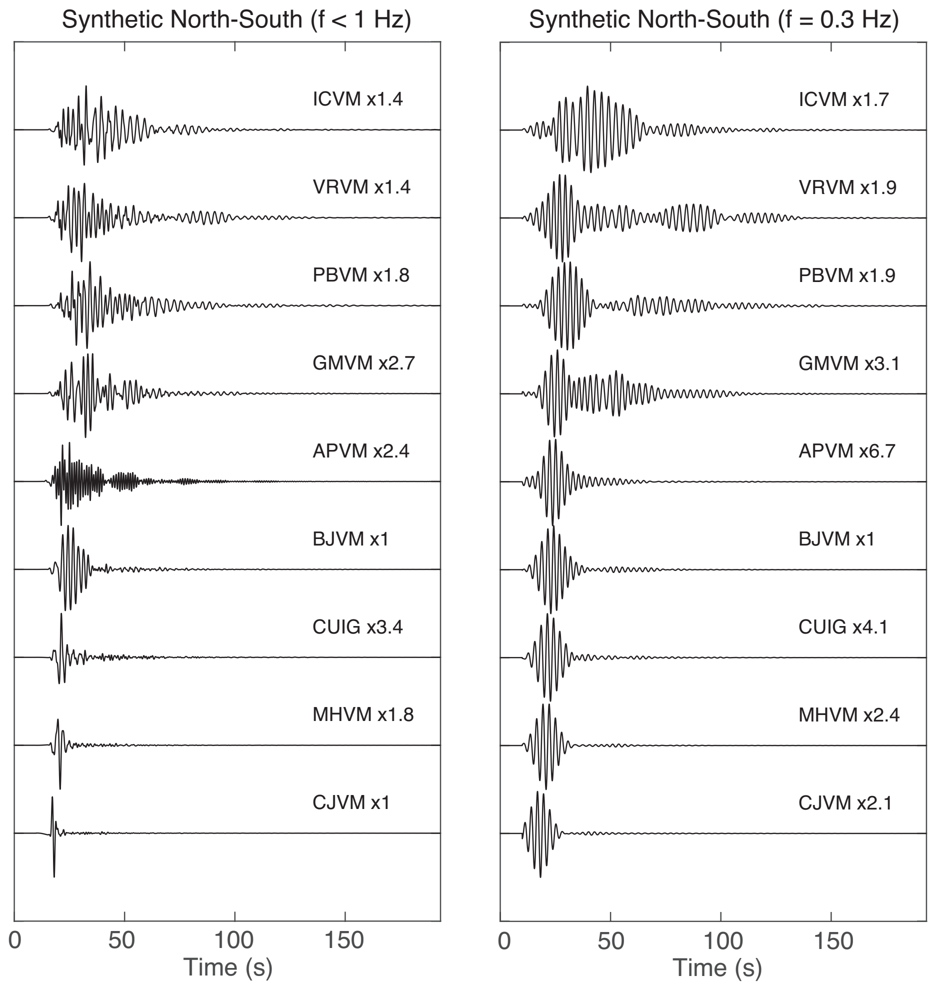

Figure A2 Synthetic velocity seismograms computed at nine broadband stations for the M3.4 earthquake with 4 km depth (Figure 1). The source is a vertical dip-slip dislocation with strike to the north and source time function shown in the inset of Figure 4. Records are aligned with the P-wave arrival. Durations of the strong shaking phases for f < 1 Hz are compared with real observations in Figure 1b.

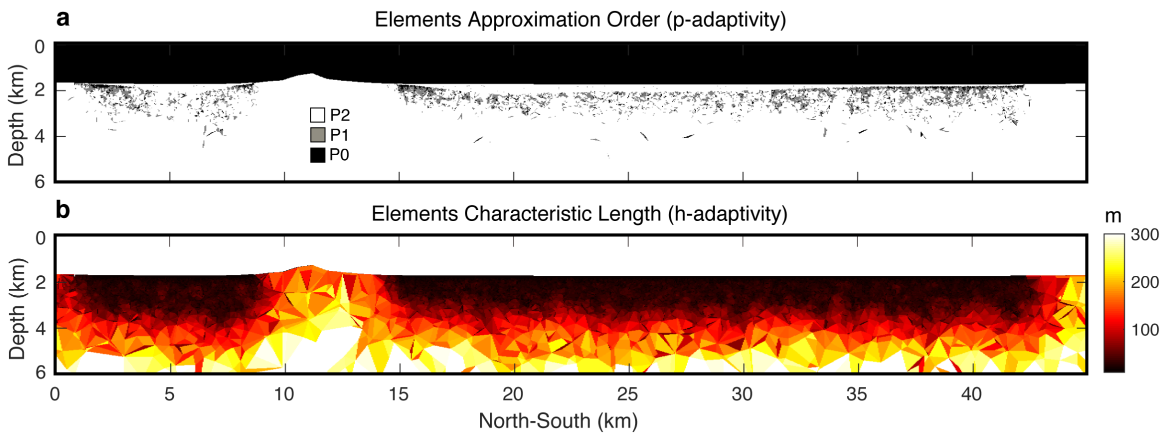

Figure A3 (a) Cross-section of the discrete model along the dashed line of Figure 6b. The distribution of the elements approximation order (i.e., p-adaptivity) is indicated with three different colors. Notice the concentration of low-order elements right below the interface with highest impedance contrasts (i.e., below the shallower basin regions close to its borders). (b) Same cross-section showing the tetrahedral mesh refinement within the basin (h-adaptivity).

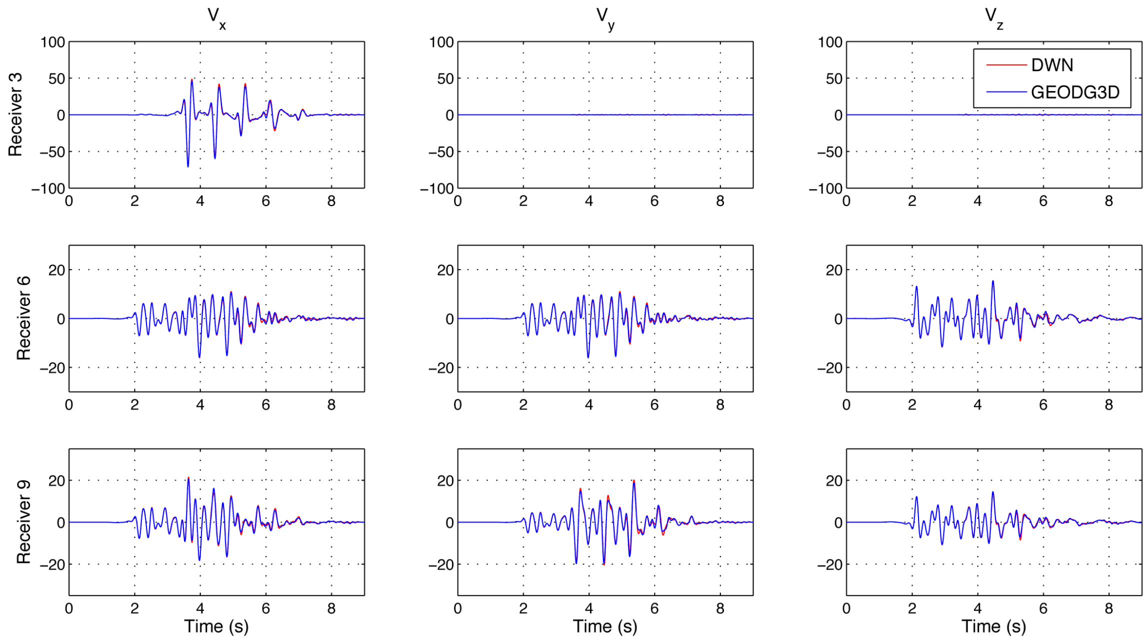

Figure A4 Comparison of synthetic seismograms (bandpass filtered between 1 and 5 Hz) computed with the Discontinuous Galerkin GEODG3D method (blue) and the Discrete Wave Number method (DWN, red) for the viscoelastic benchmark "Layer Over a Halfspace 3" (LOH3) of the Southern California Earthquake Center (SCEC) described by Day et al. (2003). Both solutions were computed with exactly the same viscoelastic modulus for three relaxation mechanisms so the signals misfit only responds to numerical approximation errors. The GEODG3D solution was computed using a P2 unstructured mesh with characteristic size of 100 m. The three receivers are located at distances from the source of about 32 times the minimum wavelength.

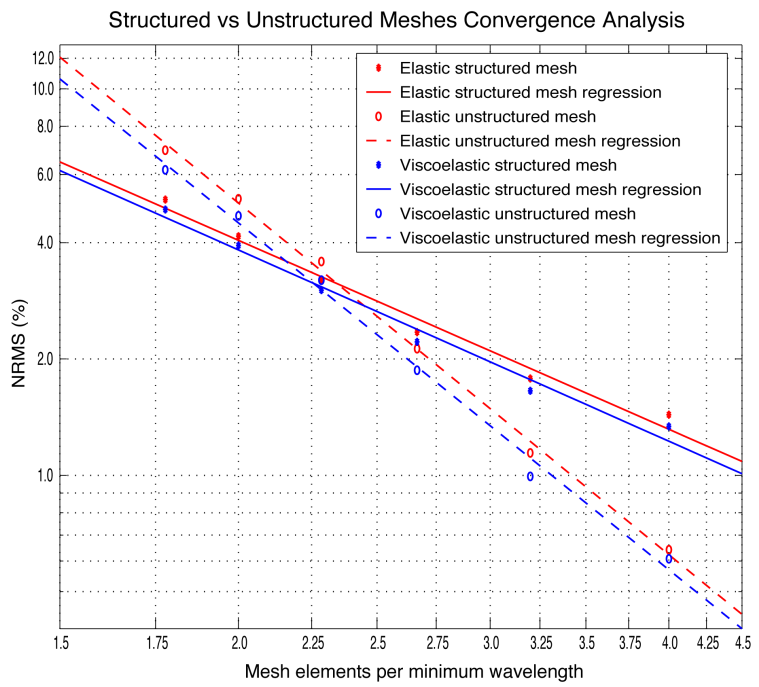

Figure A5 Convergence analysis for GEODG3D elastic (red) and viscoelastic (blue) methods considering structured (solid) and unstructured (dashed) meshes. Problems used were the LOH1 and LOH3 (Day et al., 2003) taking the DWN solutions as references. Viscoelastic solutions are always better that the elastic. Convergence rate in unstructured meshes (slope of 2.98) is significantly higher than the corresponding value for structured meshes (slope of 1.64). This is due to the numerical anisotropy induced by the regularity of elements in the structured mesh.

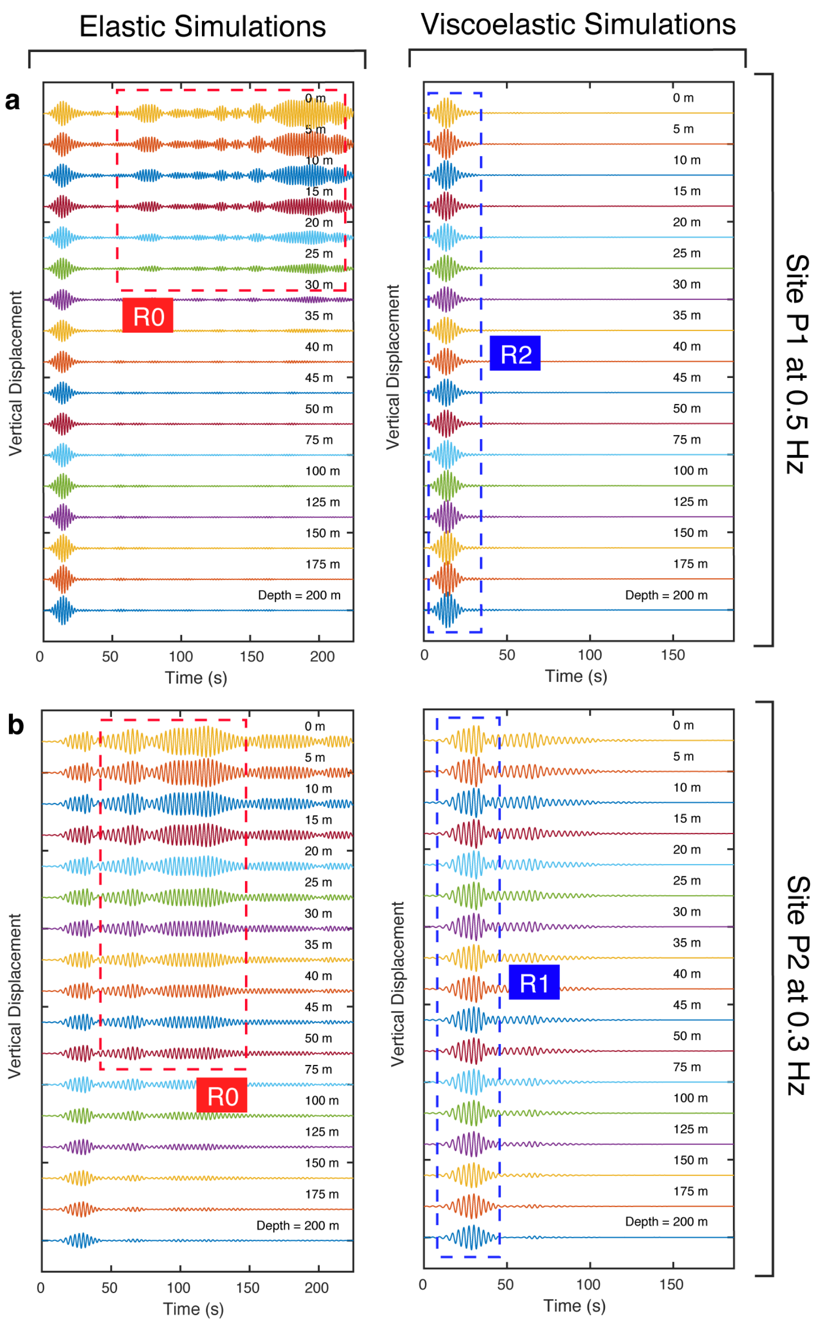

Figure A6 Synthetic borehole seismograms at sites P1 and P2 (see Figure A9) for two different frequencies. While the energy of the fundamental mode (R0) decays rapidly with depth (red rectangles), that of the first and second overtones (R1 and R2) persists along the entire depth of the borehole. Compare the amplitudes of the wave packages with the eigenfunctions of Figures 6a and 6c.

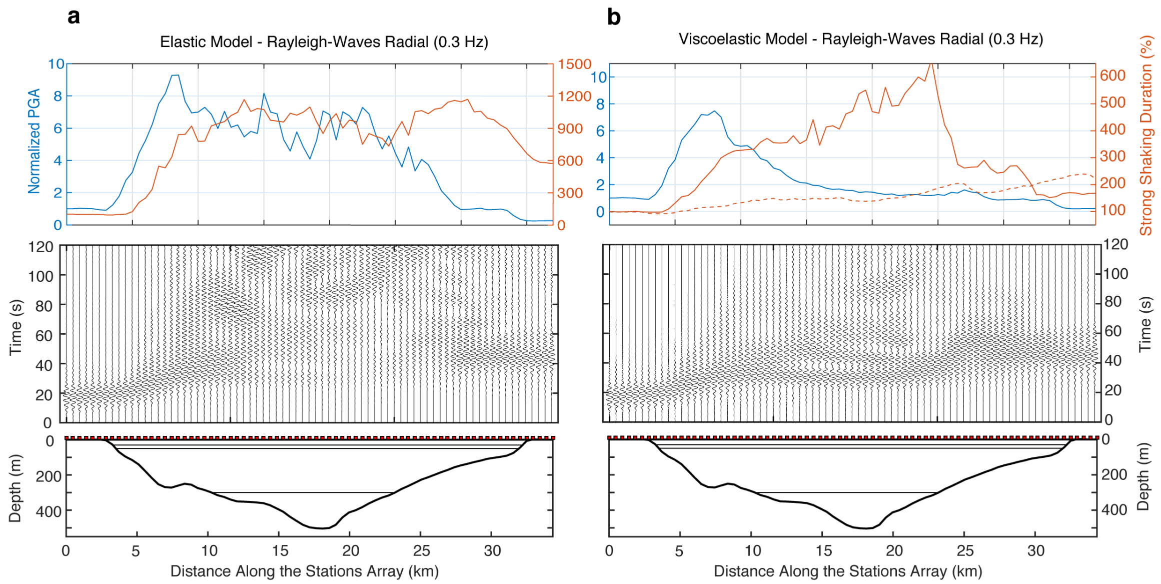

Figure A7 Same as Figure 5 but for f = 0.3 Hz.

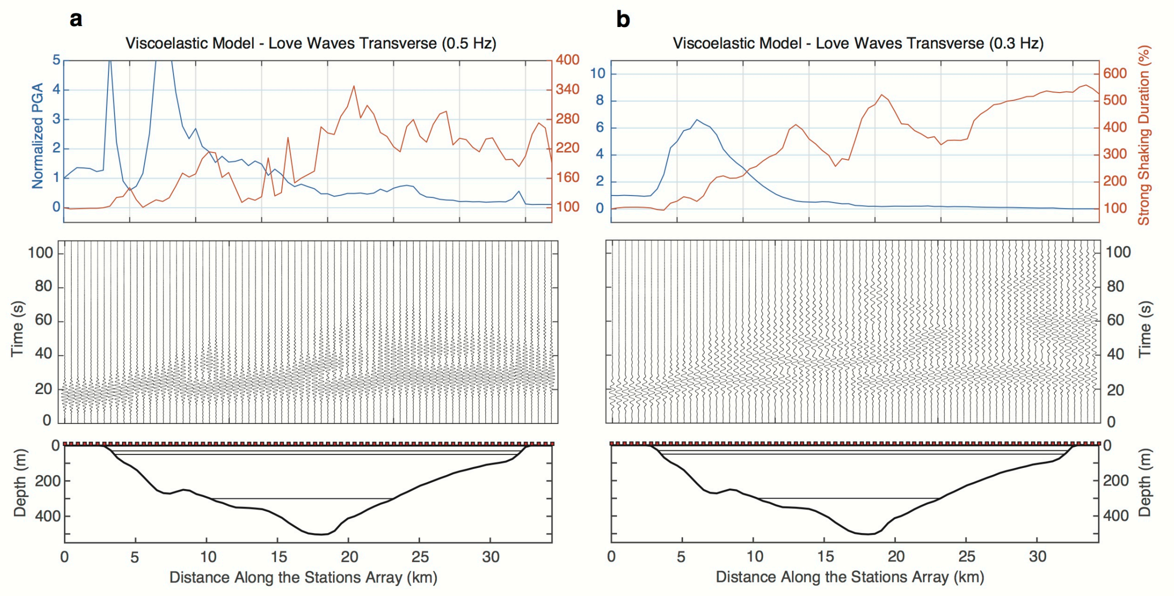

Figure A8 Same as Figure 5 but for Love waves in the viscoelastic model at (a) f = 0.5 Hz and (b) f = 0.3 Hz. This simulation corresponds to a 1.5 km depth double-couple strike-slip point source at location S6.

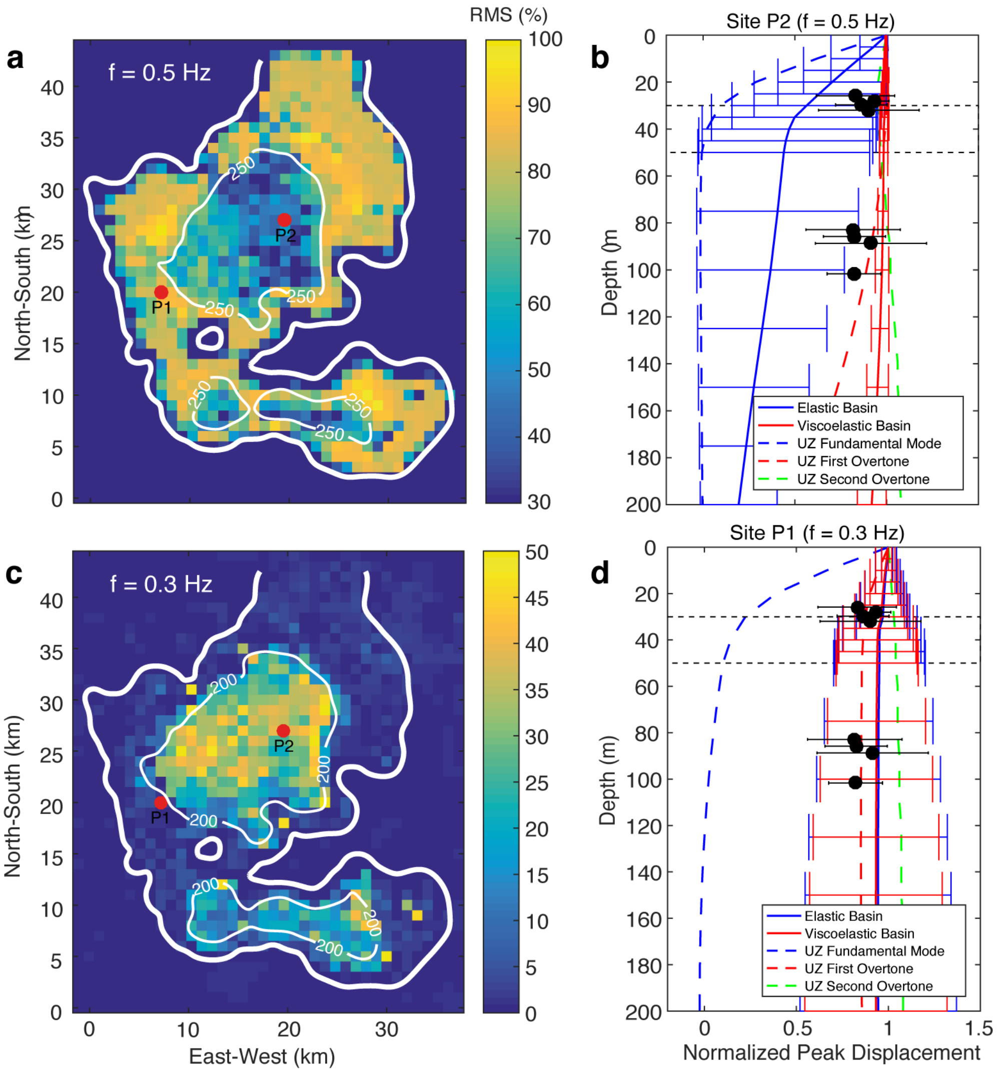

Figure A9 (a) and (c) RMS differences of the averaged (for the eight sources) elastic and viscoelastic eigenfunctions computed in the whole borehole network shown in Figure 1 for two different frequencies. White contours delineate the basin geometry at fixed depths in meters. Yellow colors depict regions where attenuation plays a major role resulting in the dominance of Rayleigh waves first overtones. (b) and (d) Same as Figure 6 but for frequencies of 0.3 and 0.5 Hz at representative sites P1 and P2. Notice the dominance of first overtones at 0.3 Hz for both elastic and viscoelastic models in shallow basin regions (i.e., at P1). This figure has been created using the Matlab software Version R2016a, http://www.mathworks.com/.

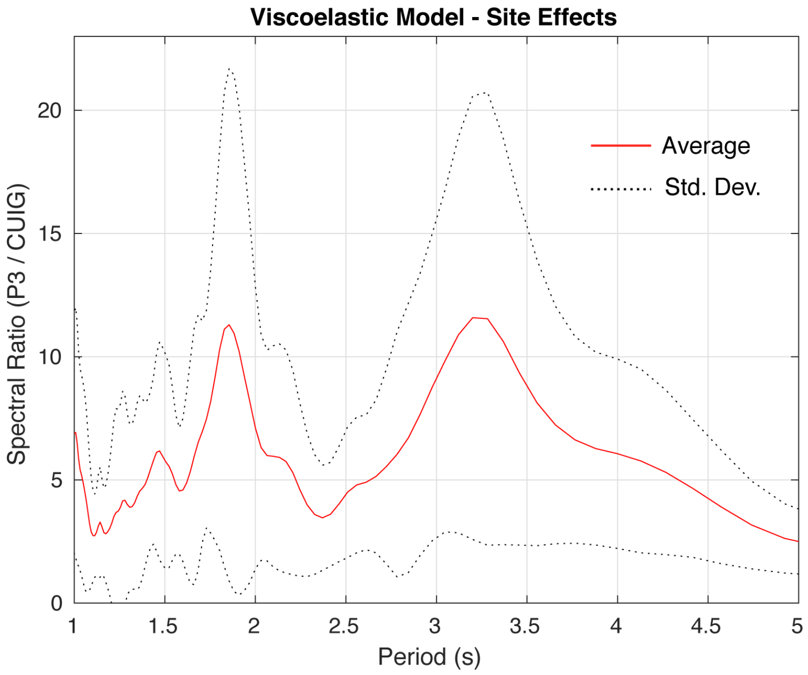

Figure A10 Average horizontal spectral amplification for the eight sources (solid red line) and standard deviations (black dotted lines) at the lake-bed representative site P3 (Figure 6) with respect to the hard-rock CUIG site.

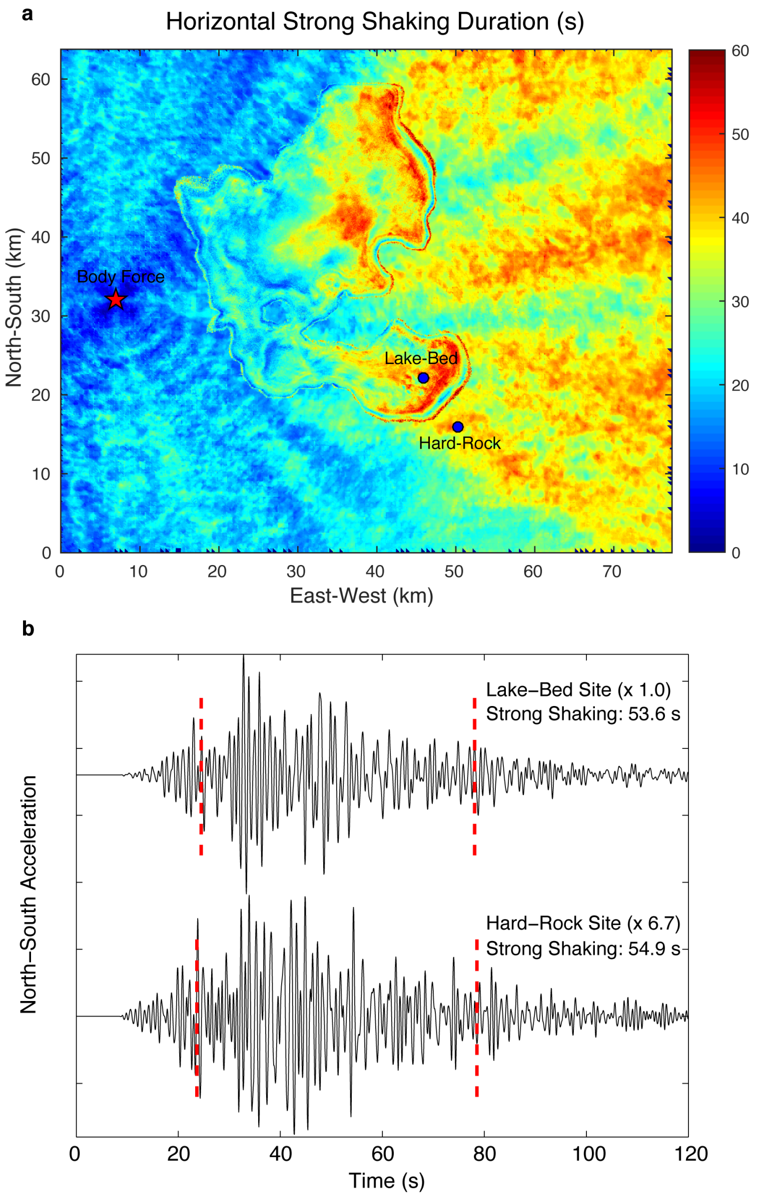

Figure A11 (a) Duration of the strong shaking phase of the Green's function due to source S6 (Figures 1 and 4) averaged for both horizontal components and f < 1 Hz. Notice the long duration of ground motions in both the sedimentary basin and the external shadow region. (b) Seismograms computed in two close sites, one in the lake-bed zone and the other at hard-rock within the seismic shadow. Red dashed lines indicate the strong shaking phase computed form the Arias intensity. Although very different in amplitude, durations of their strong phases are almost the same. This figure has been created using the Matlab software Version R2016a, http://www.mathworks.com/.
